# Cytocompatibility Assessment of L-PBF-Manufactured Zinc–Silver–Copper Alloys for Customized Biodegradable Medical Implants

**DOI:** 10.3390/jfb17030146

**Published:** 2026-03-17

**Authors:** Barbara Illing, Jacob Schultheiss, Lukas Schumacher, Evi Kimmerle-Mueller, Ariadne Roehler, Alexander Heiss, Ulrich E. Klotz, Victor O. Okafor, Stefanie Krajewski, Frank Rupp

**Affiliations:** 1 Department of Medical Materials Science and Technology, Institute of Biomedical Engineering, University Hospital Tübingen, Osianderstrasse 2-8, 72076 Tübingen, Germanyariadne.roehler@med.uni-tuebingen.de (A.R.); stefanie.krajewski@med.uni-tuebingen.de (S.K.); frank.rupp@med.uni-tuebingen.de (F.R.); 2 Department of Physical Metallurgy, fem Research Institute, Katharinenstraße 17, 73525 Schwäbisch Gmünd, Germany; heiss@fem-online.de (A.H.); ulrich.klotz@hm.edu (U.E.K.);

**Keywords:** Zn alloys, implants, additive manufacturing, laser powder bed fusion, biodegradation, ion release kinetics, cytocompatibility

## Abstract

Biodegradable zinc (Zn) has attracted increasing interest as a material for temporary implants, primarily due to its moderate degradation kinetics. In recent years, additive manufacturing of Zn alloys using the laser powder bed fusion method (L-PBF) has shown promising results. Compared to as-cast Zn alloys, it offers preferable customized solutions for patient-specific temporary biomedical implants. Due to the novelty of these printed degradable biomaterials and due to reported cytotoxic effects of Zn alloys, this study investigates additively manufactured ZnAgCu, ZnAgCuMn, and ZnAgCuTi alloys, both in as-printed and post-processed conditions, with a focus on L929 and SAOS-2 biocompatibility. In this work, we demonstrate that the increased porosity and therefore larger surface areas compared to polished Zn-alloy samples affect their biocompatibility. Minimal to no cell proliferation was observed on and near the Zn-alloy test plates after 24 h. Undiluted extracts from as-cast Zn and L-PBF-manufactured plates were initially cytotoxic to SAOS-2 cells. However, as passivation proceeded, cytocompatibility was significantly increased from day 3 onward. Zn^2+^ ion release peaked at 24 h and declined significantly from day 2 to day 10. Compared to the other Zn alloys, ZnAgCuMn exhibited the lowest cytocompatibility. Most intriguingly, 3-month surfaces exhibited reduced cytocompatibility to osteoblasts compared to freshly polished samples. The observed in vitro cytotoxicity motivates further investigation of as-printed and post-processed L-PBF-manufactured Zn alloys, aiming to develop novel surface modification strategies to mitigate the initial ion burst responsible for reduced cytocompatibility and to adjust and tailor the overall degradation kinetics to physiologically tolerable levels tailored to the intended clinical application.

## 1. Introduction

The development of biodegradable materials for temporary medical implants has gained momentum as an alternative to permanent metals, which often require additional surgical removal. Biodegradable implants are designed to support damaged bone or tissue during healing and gradually dissolve as the tissue regains functionality. With an increasing demand for biodegradable materials in orthopedic applications, such as fracture fixation, the exploration of novel alloy systems has become crucial.

Biodegradable implant materials must exhibit high mechanical stability, controlled degradation kinetics, ensure tissue compatibility, and minimize cytotoxic effects. Considerable research efforts have been directed toward the investigation of biodegradable magnesium (Mg) and iron (Fe) alloys as potential implant materials over the past decades [1,2,3,4,5,6,7], but these have limitations. Fe alloys possess high ductility but suffer from insufficient degradation kinetics, resulting in insoluble by-products remaining in the tissue [8,9]. Mg alloys, on the other hand, exhibit some favorable mechanical properties. However, their rapid corrosion rate, coupled with concomitant mechanical instability, surface pitting corrosion and hydrogen evolution, limits their use in most medical applications [10,11].

Zinc (Zn) has emerged as a promising material for bioresorbable implants due to its high cytocompatibility and essential role in physiological processes, making it an attractive material for biomedical applications. Additionally, the recommended daily intake of Zn for adults typically ranges from 8 to 11 mg/day according to established nutritional guidelines [12]. Zn is abundant in bones and muscles and plays a crucial role in cellular function, immune response, wound healing, and bone mineralization [13,14,15].

Preliminary studies and in vivo investigations of pure zinc stents have demonstrated corrosion rates that align with the healing process, showing no significant inflammatory responses or thrombosis [16,17,18,19,20].

However, pure Zn and some of its alloys exhibit limitations, including insufficient mechanical properties, limited thermal stability, slow corrosion rates, and low creep resistance [21]. To address these limitations, effects of alloying Zn with silver (Ag), gold (Au), copper (Cu), magnesium (Mg), calcium (Ca), strontium (Sr), manganese (Mn) and lithium (Li) to enhance the mechanical properties by solid solution and precipitation strengthening, improve anti-aging properties or adjust corrosion rates, were studied [22,23,24,25,26,27,28,29]. Studies on the corrosion behavior of Zn alloys in simulated physiological conditions have revealed their ability to degrade gradually at rates comparable to tissue healing [19,30,31,32,33,34,35]. These alloys exhibit tunable degradation rates, allowing for tailored implant lifetimes based on specific clinical requirements. Alloying with these elements has not only been shown to improve mechanical properties. Furthermore, Mg, Sr, and Mn also enhance the cytocompatibility of Zn alloys [36,37,38].

In recent years, doubts have been raised about Zn as biocompatible in view of the concentrations released in vitro. Numerous studies have suggested that only diluted extracts of Zn alloys exhibit sufficient biocompatibility. However, definitive conclusions about the cytotoxicity of Zn alloy extracts remain challenging due to significant variability across studies. These differences concern variations in a few experimental conditions, such as alloy composition, cell cultures, material surface areas used for extract generation, immersion media, extraction temperature, and various test methods and chemicals for determining the proliferation of the respective cells [39]. Despite these inconsistencies, an increasing number of more recent studies indicate that undiluted extracts may exhibit cytotoxic effects on cell cultures in vitro.

Additive manufacturing by laser powder bed fusion (L-PBF) has emerged as a key technology in digital manufacturing [40]. L-PBF enables the bottom-up creation of complex components, eliminating the need for molds and thus facilitating the production of customized orthopedic implants with intricate geometries tailored to patient-specific anatomies. The development of personalized biodegradable implants enhances the overall biomechanical compatibility, reducing the risk of complications such as implant loosening or discomfort. This ultimately benefits patients by providing a more effective treatment option. However, manufacturing Zn-based parts presents challenges due to Zn’s low melting and boiling points, which can lead to evaporation, formation of pores and poor process stability [41,42,43].

Due to the challenges mentioned above, much attention has been paid to the porosity, degradation behavior and mechanical stability of printed Zn alloys, while the focus on biocompatibility was less pronounced. To date, there are only a few studies focusing on the cytotoxicity of Zn alloys manufactured by L-PBF [44,45]. Accordingly, the present work closely examines the in vitro biocompatibility of L-PBF-manufactured Zn alloys, using both direct contact and indirect extract tests, considering both unprocessed, as-printed and post-processed Zn alloys.

In continuation of our previous work, the ZnAgCu alloy system was selected based on thermodynamic calculations [22,33,46,47]. Ag and Cu were added not only to improve mechanical properties [48,49], but also to impart antibacterial activity. Mn was specifically selected as an alloying partner, as the literature reports beneficial effects of Mn on mechanical properties and biocompatibility [36,43,50,51,52]. Lin and coworkers investigated the potential of Zn-1Cu-0.1Ti alloys for orthopedic applications. They observed the formation of three phases, including a η-Zn phase, an ε-CuZn_5_ phase, and an intermetallic phase of TiZn_16_. The alloy showed good mechanical properties and good biocompatibility [53]. Finally, three distinct L-PBF-manufactured Zn alloys were included in this study: ZnAgCu, ZnAgCuMn, and ZnAgCuTi. Beyond process optimization and degradation studies, this work primarily focused on biocompatibility assessment using L929 mouse fibroblasts and SAOS-2 human osteogenic sarcoma cells.

## 2. Materials and Methods

### 2.1. Sample Manufacturing

#### 2.1.1. Alloying and Atomization

Twelve kilograms of each Zn-based alloy were cast in a protective argon (Ar) atmosphere at Meotec GmbH (Aachen, Germany) using the pure elements Ag, Zn, and Ti, as well as the master alloys Cu-42Zn (Wieland AG, Ulm, Germany) and Cu-30Mn (Indutherm VTC 200 V/Ti casting machine, Walzbachtal, Germany). The composition of the alloys was confirmed by X-ray fluorescence analyses (Fischerscope XRAY XDV-SDD50, Helmut Fischer GmbH, Sindelfingen, Germany). L-PBF powders were produced by gas atomization and subsequent air classification (10–45 µm) by Indutherm GmbH. Powder size distribution was assessed by dynamic image analysis (DIA, Camsizer X2, Microtrac Retsch GmbH, Haan, Germany), powder morphologies were investigated by field emission scanning electron microscopy (FE-SEM, Auriga 60, Zeiss, Oberkochen, Germany) and the chemical composition of the powders was assessed by inductively coupled plasma optical emission spectrometry (ICP-OES, 5110, Agilent Technologies Inc., Santa Clara, CA, USA). Alternatively, the chemical composition was also assessed by XRF.

#### 2.1.2. Additive Manufacturing

Based on the process map derived from a principal component-based optimization approach, only small changes in laser parameters were necessary for a robust L-PBF process with ZnAgCu powders. Based on a previous systematic investigation on L-PBF with Zn alloy [54], experimental plates (10 × 10 × 2 mm^3^) were manufactured on a Concept Laser Mlab R machine (Concept Laser, Lichtenfels, Germany) with a volumetric energy density of VED = 47 J/mm^3^. The VED of 47 J/mm^3^ in this study was derived from the Zn-4Al-1Cu optimization strategy presented in Heiss et al. [54], where a processing window between 40 and 60 J/mm^3^ was found. The solidified melt pools were visualized by dark field optical microscopy (DF-OM). As the surface area is critical for the subsequent assessment of the cytotoxicity, the dimensions ((10.5 ± 0.3) mm × (10.2 ± 0.1) mm × (2.2 ± 0.1) mm) of each quadratic L-PBF plate were checked.

The investigated alloys were produced exclusively by L-PBF. Comparable alloys manufactured by conventional processing routes were not included except for a Zn reference sample. Additionally, reference samples of the same dimensions made of rolled Zn (Zn, 99.99% Zn, HMW Hauner GmbH, Röttenbach, Germany), a copper (Cu) sheet, and titanium grade two disks (Ti, Straumann AG, Basel, Switzerland) were included as controls.

#### 2.1.3. Pretreatment of Zn Alloy Samples

After manufacturing, all sample surfaces were thoroughly cleaned under distilled water with a brush to remove any loose powder particles that may still be adhering (hereinafter referred to as “untreated”).

The surfaces of the L-PBF-manufactured samples were significantly enlarged due to the spherical structure of the powder feedstock. To achieve the surface size prescribed by the DIN standard ISO 10993-12: 2012 [55] for the cytotoxicity extract tests and to investigate the effects of the surface enlargement due to the manufacturing, half of the samples were ground with SiC sandpaper (P1200 CarbiMet, BUEHLER, Lake Bluff, IL, USA) until a smooth surface was achieved. Both the original L-PBF surfaces and the ground surfaces were compared in the indirect cytotoxicity test. At the time of this biological test, the plates had been ground 1–3 days before (Table 1).

To clarify whether the degree of aging of the alloys also plays a decisive role in toxicity, further experiments were carried out. All L-PBF-manufactured plates were ground to achieve a comparable surface area. The ground plates were stored under ambient conditions for 3 months in a sterile bench. After this aging (referred to as “aged polished”), half of the plates were polished again to obtain a non-aged, freshly polished surface directly before the experiment (“new polished”) (Table 1).

All test plates were cleaned and disinfected with 70% ethanol for 10 min in an ultrasonic bath before each biological experiment.

### 2.2. Sample Characterization

#### 2.2.1. µCT Examination

Fifteen random Zn alloy plates (3 × 5) were investigated by computed tomography (µCT, 300 kV V|TOME|X L 450, GE Sensing & Inspection GmbH, Wunstorf, Germany). The plates were analyzed for density and pore distribution using the software VG Studio Max 3.5.2 (Volume Graphics GmbH, Heidelberg, Germany). The density at the boundary and the local thickness [56] were assessed by means of binary stacks in ImageJ (ImageJ 1.54p). The fractal dimension was assessed with the box-counting method using the BoneJ2 plugin. Respective linear regressions had an R^2^ > 0.99.

#### 2.2.2. Scanning Electron Microscopy

The surface morphology of each sample group and the morphology and location of the cells on the test surfaces were characterized by SEM (LEO 1430, Zeiss, Oberkochen, Germany) with an accelerating voltage of 5 kV using the secondary electron detector. Elemental composition was measured with an energy dispersive X-ray spectroscopy (EDX) detector (EDR 288/SPU2 Röntec, Berlin, Germany) at an accelerating voltage of 10 kV. For this purpose, the samples with cells were fixed in 2% (*v*/*v*) glutaraldehyde overnight, followed by ascending ethanol dehydration. Subsequently, the samples were critically point-dried (Polaron E3100, Judges Scientific plc, London, UK). Before microscopic analyses, only the samples with attached cells were sputtered (BAL-TEC SCD050, Balzers, Lichtenstein) with Au-20Pd.

The Zn alloy samples, which had been stored in McCoy’s medium for 10 days to prepare extracts and investigate time-dependent ion release, were thoroughly rinsed with deionized water and air-dried before subsequent electron microscopic analysis. This step was taken to prevent the formation of surface crystals from dried medium residues.

#### 2.2.3. Assessment of Roughness

The topography of the different surfaces was determined by confocal microscopy (MarSurf CM Explorer, Mahr GmbH, Göttingen, Germany) and the roughness was analyzed using MountainsMap (Version 9.1.9957, Digital Surf SARL, Besançon, France). For this purpose, three plates of each surface modification were evaluated. On each plate, six different areas of 800 × 800 µm^2^ were scanned using a 20× objective (0.6 numeric aperture). For analysis, first, the shape was removed using the software (LS-Poly2). With an L-Filter (λc, high-pass, Gauss 0.05 mm), roughness was separated from waviness, and an S-Filter (λs, low-pass, Gauss 300:1 compared to the L-Filter) was applied to remove possible noise. Arithmetic mean roughness heights (Sa) and the developed interfacial area ratio (Sdr) (percentage of the additional surface area compared to the planar geometric area, expressed as a percentage of the geometric area, an Sdr value of zero corresponds to a completely plane surface) were calculated for each surface modification.

### 2.3. Biocompatibility Testing

#### 2.3.1. Cultivation of Cells

To test the biocompatibility, the mouse fibroblast cell line L929 (Leibniz Institute DSMZ-German Collection of Microorganisms and Cell Cultures GmbH, Braunschweig, Germany), cultured in Dulbecco’s modified Eagle medium (DMEM, Thermo Fisher Scientific Inc., Waltham, MA, USA) supplemented with 10% fetal bovine serum (FBS SUPERIOR, Bio&SELL GmbH, Feucht, Germany) and 1% L-glutamine (Thermo Fisher Scientific Inc.) was used. In addition, the human primary osteogenic sarcoma cell line SAOS-2 (Leibniz Institute DSMZ-German Collection of Microorganisms and Cell Cultures GmbH), cultured in McCoy’s 5A medium (Sigma-Aldrich Chemie GmbH, Steinheim, Germany) supplemented with 15% FBS (Bio&SELL GmbH) and 1% L-glutamine (Thermo Fisher Scientific Inc.), was used. To avoid contamination, 1% penicillin and streptomycin (Penicillin/Streptomycin [+] 10,000 Units/mL penicillin [+] 10,000 μg/mL streptomycin, Thermo Fisher Scientific Inc.) were added to both culture media. Cells were cultured under standard cell culture conditions (37 °C, 5% CO_2_ and 95% relative humidity).

#### 2.3.2. Direct Cytocompatibility Testing

To assess the possible toxicity of the alloys in direct contact with the cells, the L-PBF-manufactured plates were affixed with dental wax to the center of a well of a six-well plate and 30,000 cells/cm^2^ (285,000 cells in total) in 5 mL of cell culture medium were added. After 24 h of incubation, the cell growth in the well and around the plates was microscopically analyzed (Nikon Optiphot-2 fluorescence microscope with EOS 550D Utility software, Nikon, Japan).

#### 2.3.3. Direct Cytotoxicity (Live-Dead Staining)

To visualize the vital status of the cells exposed to the different samples (direct cytotoxic test), a live-dead staining was performed using the Live/Dead BacLightTM Viability kit (Thermo Fisher Scientific Inc.) containing SYTO9 (green stain for live cells) and propidium iodide (red stain for dead cells) according to the manufacturer’s instructions. Stained cells were documented through fluorescence microscopy (Nikon Optiphot-2).

#### 2.3.4. Cytocompatibility Extract Testing

An extract test with L929 and SAOS-2 cells to evaluate the biocompatibility of the L-PBF-manufactured Zn alloys was performed in accordance with ISO 10993-12: 2012 [55]. Titanium grade 2 (Ti) was used as the negative control, while pure Cu extract was used as the positive control.

For the preparation of the extract, the samples were exposed to the respective cell culture medium for 24 h at an extraction ratio of one sample plate per ml at 37 °C and 5% CO_2_. A total of 10,000 cells per well were seeded in a 96-well plate with the corresponding cell culture medium. After 24 h of incubation, the cell culture medium was replaced with three different extract concentrations: undiluted 100% (150 µL), 33% (50 µL) and 10% (15 µL) extract. Cell proliferation was evaluated using a BrdU ELISA test (Sigma-Aldrich Chemie GmbH, St. Louis, MO, USA) according to the manufacturer’s specifications. Proliferation was determined with the thymidine analog BrdU (5-bromo-2′-deoxyuridine) after it was incorporated into the newly synthesized DNA and then measured photometrically with an anti-BrdU antibody after 24 h of exposure to the different extract concentrations. Each experiment was conducted independently four times with four replicates per test.

#### 2.3.5. Time Dependency of Ion Releasing

Furthermore, undiluted extracts were tested for cytotoxicity and analyzed by ICP-OES over a period of 10 days to detect changes in ion concentration over time. For this purpose, the extract medium (McCoy’s supplemented with 15% FBS, 1% L-glutamine, and 1% penicillin and streptomycin, at 37 °C and 5% CO_2_) was collected daily from the sample plates and renewed for 10 days. The cytocompatibility extract test was performed with SAOS-2 cells incubated for 24 h with extracts obtained on day 1, 3, 5, and 10 after incubation. For the ICP-OES analysis, all extracts obtained from the 10 days were analyzed (as described in Section 2.4.1). Tests were performed with untreated alloys and alloys polished three days before use.

#### 2.3.6. Untreated, New Polished and Aged Surfaces in Extract Testing

To determine the impact of aging of the Zn alloys on the cytocompatibility, untreated (as printed) L-PBF-manufactured sample extracts taken after 24 h of incubation in McCoy’s were compared with extracts of freshly polished and aged polished samples (aged under ambient room conditions for 3 months) in the extract test as described in Section 2.1.3 (Pretreatment of Zn alloy samples).

### 2.4. Corrosion

#### 2.4.1. Ion-Release Concentration

The concentrations of ions released from the samples were determined by ICP-OES (Avio 220 Max, Perkin Elmer, Shelton, CT, USA). The extracts were analyzed for five elements (Zn, Ag, Cu, Mn, and Ti), each being analyzed with its corresponding most sensitive wavelength. For the measurement, the extracts were diluted 1:3 with 2% *v/v* HNO_3_. As a control group, in addition to the extracts, fresh McCoy’s medium was diluted in the same way and analyzed accordingly. All calibration standards were also made with fresh and diluted McCoy’s medium. The mean of the measured concentrations of the control was calculated and subtracted from the measured concentrations of the samples, and finally multiplied by three to obtain the undiluted concentration in the extracts. Five sample plates per alloy were measured (*n* = 5).

#### 2.4.2. Potentiodynamic Polarization

To supplement the corrosion rate data from the ion-release measurements, corrosion rates were also calculated by the Tafel extrapolation method from potentiodynamic polarization curves [57,58]. The polarization was carried out with a VersaSTAT 3 potentiostat (AMETEK Scientific Instruments, Oak Ridge, TN, USA) using a three-electrode setup. The software used was VersaStudio Version 2.62.2 (AMETEK Scientific Instruments). An Ag/AgCl 3M KCl reference electrode (RE) (Metrohm, Herisau, Switzerland) and a Pt-counter electrode (CE) (Metrohm, Herisau, Switzerland) were used. The square-shaped, additively manufactured samples served as the working electrode and were connected via a customized sample holder, exposing an area of 0.503 cm^2^ to the medium. The measurements were conducted in 72 mL of Dulbecco’s Phosphate Buffered Saline (Thermo Fisher Scientific Inc.) without Ca^2+^ and Mg^2+^ (DPBS^−−^) with 8 mL FBS at 37 °C, to avoid potential adsorption of cell culture medium components onto the electrode surface when using McCoy’s. In preparation for the measurements, the samples were cleaned with a soft brush while being rinsed with deionized water. They were then cleaned with ethanol (70%) for 5 min at room temperature using an ultrasonic bath and stored under ambient conditions for at least 72 h to dry. Before each measurement, the samples were rinsed first with acetone, then with isopropanol, and finally with deionized water.

After measuring the open circuit potential (OCP) until stabilization, a potentiodynamic polarization was performed from −0.2 V (vs. OCP) to −0.5 V (vs. RE) with a scan rate of 3 mV/s. Corrosion rates were calculated from the corrosion current determined by Tafel-extrapolation within the software. Five samples per alloy were measured.

### 2.5. Statistical Analysis

Unless stated otherwise, data are presented as mean ± standard deviation (SD). First, the normal distribution of the data was assessed using the Shapiro–Wilk test. The data of the indirect cytotoxicity experiments were normalized to the results of cells grown without the extract in the cell culture medium. Each cytotoxicity experiment was performed independently four times (in one experiment, the experiment was performed eight times as stated in the figure legends) with four samples per alloy. The Student’s *t*-test was used to compare means between two groups. Statistically significant differences between means of three or more groups were determined using one-way analysis of variance (ANOVA) with subsequent post hoc Tukey’s test if necessary. All statistical analyses were performed using the statistical software package GraphPad Prism (Version 9.4.1, GraphPad Software, San Diego, CA, USA). Statistical significance was defined as *p* < 0.05.

## 3. Results

### 3.1. Characterization of Alloys and Powders

#### 3.1.1. Characterization of Powders

**Figure 1:**
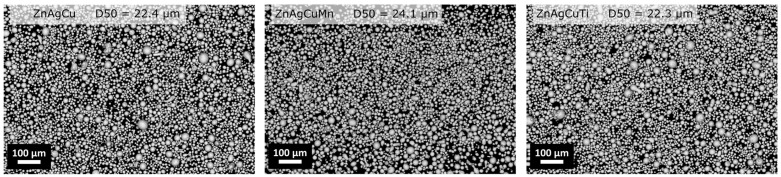
SEM images of the Zn alloys with D50 value assessed DIA.

The chemical compositions of the cast rods, the resulting powders and the final additively manufactured parts are compiled in Table 2. The SEM images in Figure 1 show the powder morphology of the three atomized powders. The median of the particle size distribution (D50) ranges between 22.3 µm and 24.1 µm.

#### 3.1.2. µCT Examination of Plates

**Figure 2:**
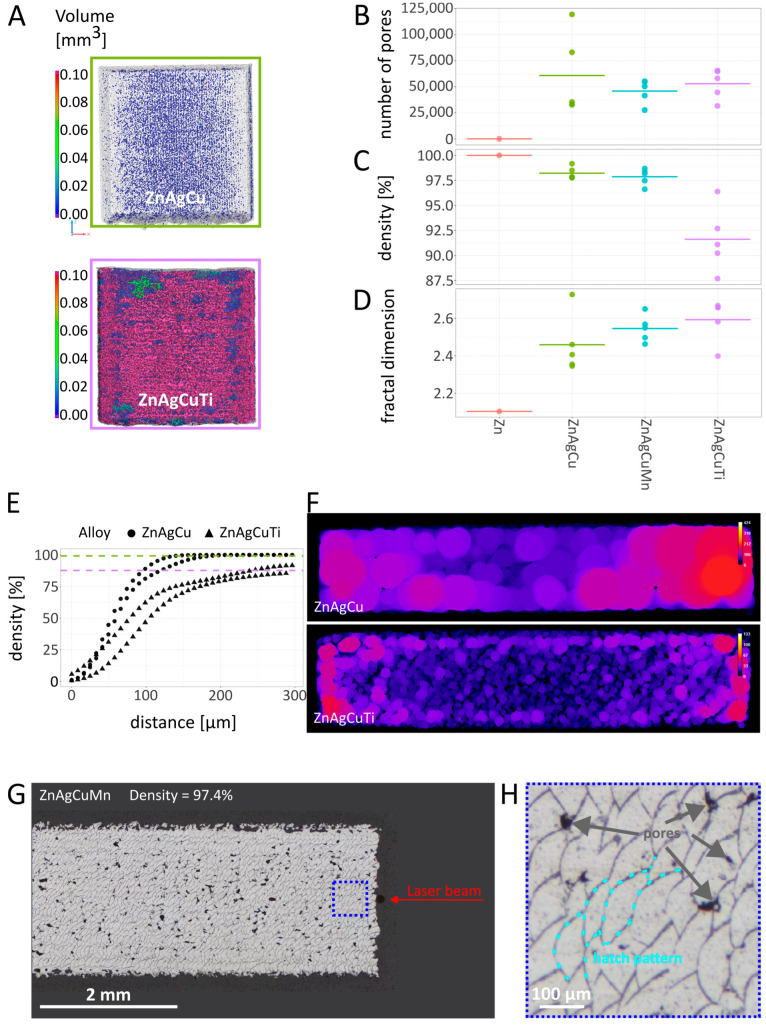
Random plate samples have been routinely analyzed by µCT. The dimensions of the plates are 10 × 10 × 2 mm^3^ (see Methods). Rolled pure Zn served as a reference. (**A**) Translucent visualization of the plate with the highest (BoB) and the lowest density (WoW), revealing the size and the distribution of the pores. (**B**) The number of pores is roughly in the same range, (**C**) density distribution of the plates, (**D**) fractal dimensions. (**E**) density evaluation of the boundary region for both sides of the two plates (BoB and WoW), the dashed lines indicate the respective total part densities, (**F**) evaluation of the local thickness as a measure of local density, ZnAgCu has a high density, whereas ZnAgCuTi has a dense boundary region but a porous core. (**G**) Dark field optical micrograph (DF-OM) of a metallographic cross-section of a ZnAgCuMn plate. The enlarged blue area (**H**) shows pores and the laser hatch pattern, i.e., the solidified melt pools. No secondary phase was observed.

Figure 2A shows the two plates with the highest (best of best, BoB) and the lowest density (worst of worst, WoW) of all plates investigated by µCT. While the number of pores is roughly the same for all three alloys (Figure 2B), ZnAgCuTi shows a lower density of 91.6% compared to ZnAgCu and ZnAgCuMn with 97.5%, indicating that the volume of the pores is considerably higher (Figure 2C). The fractal dimension reflects the enlargement of the surface by pores and protrusions (Figure 2D), Zn < ZnAgCu < ZnAgCuMn < ZnAgCuTi. Obviously, as-rolled pure Zn is much smoother than the as-printed (untreated) plates.

Evaluation of µCT data with regard to local density (Figure 2E,F) as well as optical microscopy (OM) on metallographic cross sections (Figure 2G) indicates that the boundary zone of the plates tended to be denser than the core. This is related to the L-PBF strategy, where a contour line is laser-written before hatching the area inside, resulting in a rather compact surface zone. Figure 2E represents a density profile from both quadratic surfaces towards the core for the two plates shown in Figure 2A. Due to the different degrees of porosity, the density increase is not sudden but gradual until the respective approximate levels of part density are reached (dashed lines). Figure 2F is derived from cross sections perpendicular to the build direction (mid-height), showing colored blobs indicating the local thickness. As ZnAgCu contains only a few pores, the local thickness is high, and the difference between the core and boundary is negligible. In contrast, the ZnAgCuTi sample is characterized by a rather dense boundary region and a porous core, i.e., a low local thickness. Figure 2G,H shows a cross-section of a ZnAgCuMn plate. Pores and the borders of the solidified melt pools are clearly visible in this alloy.

#### 3.1.3. Surface Characterization with SEM

**Figure 3:**
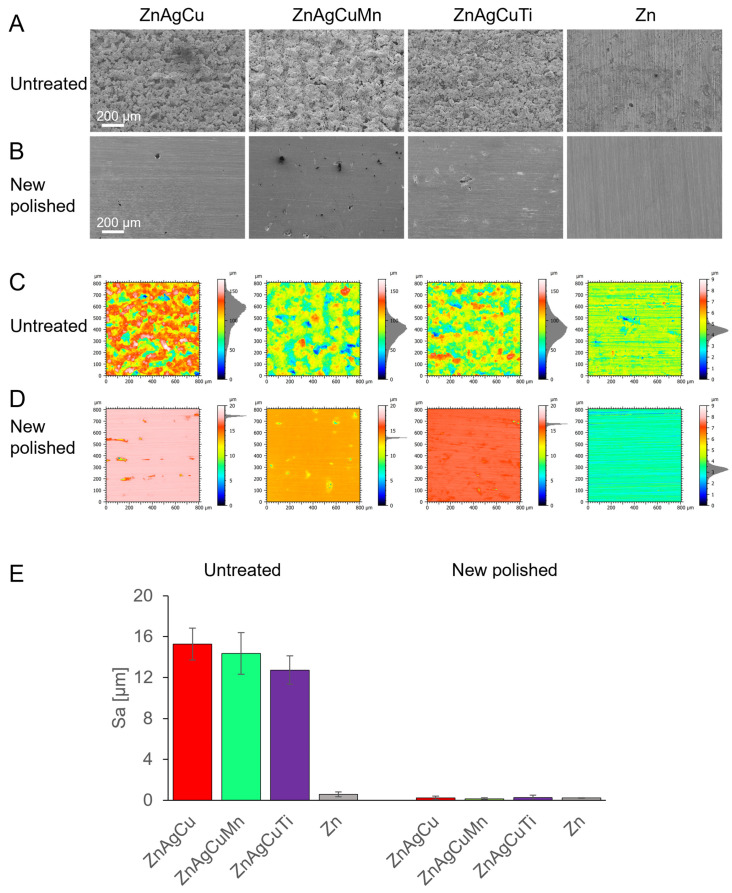
Surface characteristics: SEM images of the various L-PBF-manufactured alloys. (**A**) Untreated alloy surfaces, (**B**) plates in “New polished” condition, and (**C**,**D**) exemplary representation of the surface roughness map of a sample plate. The mean values of Sa are shown in (**E**). A rolled-Zn plate (Zn) was included as a comparison control (*n* = 3).

In agreement with the CT data (Figure 2), SEM images of the L-PBF-manufactured Zn alloys revealed that the surface of the samples exhibited significant roughness in comparison to the smooth surface of the rolled pure Zn reference plates (Figure 3A).

Upon grinding the alloy surfaces to a smooth finish (Figure 3B), it was observed that the metal powder of the L-PBF-manufactured samples had fused together effectively, forming a solid body beneath the surface. Only a few cavities, identifiable as dark spots, were remnants from the original printed surface.

#### 3.1.4. Roughness

As-printed parts are typically characterized by a much higher surface roughness compared to rolled counterparts. This appearance was confirmed by our confocal measurements (Table 3, Figure 3C), and the surface roughness (Sa) could be drastically reduced by polishing (Figure 3D,E). However, by confocal analyses, the surface area of the untreated samples, as indicated by Sdr values, was most likely underestimated due to their porous nature and the presence of voids between and beneath the spheres. The unpolished L-PBF-manufactured plates had an up to 4.4 times larger Sdr surface area compared to the respective polished specimen. Metallographic cross sections along the build direction were prepared to evaluate the surface quality and the bulk density, which ranged, in estimation according to CT data, between 92% and 99.5% (Figure 2G,H).

### 3.2. Cytocompatibility and Ion Release

#### 3.2.1. Direct Cytotoxicity

**Figure 4:**
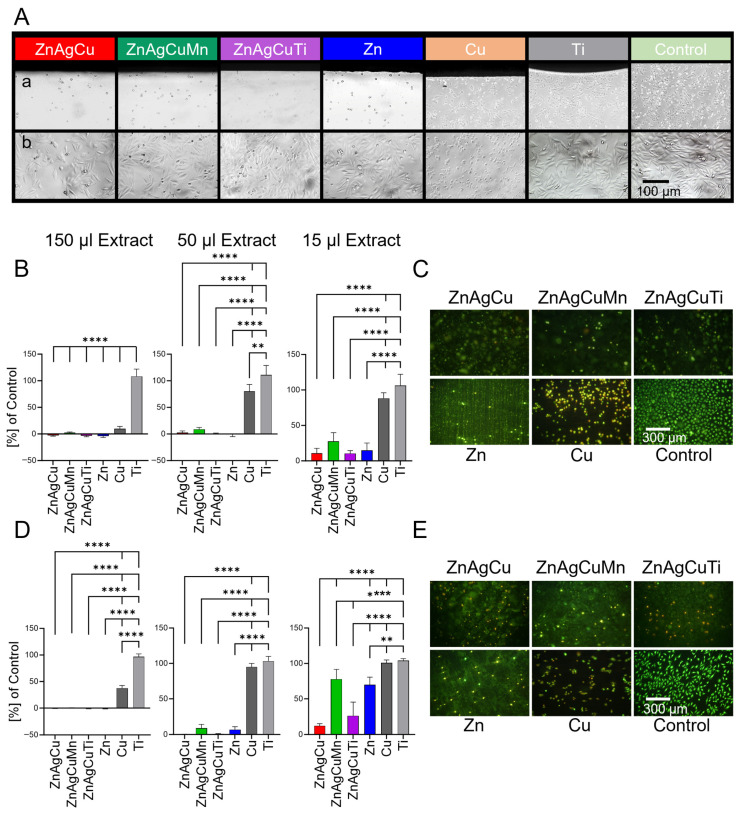
(**A**) Representative images of the direct cytocompatibility contact test with SAOS-2 cells are presented. The top row (**a**) shows the tested sample plates (black) at the top of the image, with cells growing in direct contact on the bottom of the six-well plate (five times magnification). (**b**) The cell density at a 12 mm distance from the test plates (ten times magnification). (**B**,**D**) Three different extract concentrations (undiluted, diluted 1:3 and diluted 1:10) from the three L-PBF-manufactured Zn alloys (ZnAgCu, ZnAgCuMn, and ZnAgCuTi), rolled Zn, Cu, and Ti controls were tested, and the proliferation of L929 (**B**) and SAOS-2 (**D**) cells was determined after 24 h. (**C**,**E**) Live/dead staining of L929 (**C**) and SAOS-2 (**E**) cells growing directly on top of the samples is shown. Red and yellow staining indicate dead or dying cells, respectively, while living cells appear green. Each test was carried out four times with four replicates per sample. Shown are the mean values with their corresponding standard deviations. All results were normalized to control cells grown in cell culture medium without extract (proliferation equals 100%). To calculate statistical significance, a one-way ANOVA followed by a Tukey test was carried out (** *p* < 0.01 and **** *p* < 0.0001).

The direct cytotoxicity test involved adding SAOS-2 osteoblasts and L929 fibroblasts directly to the test plates, followed by microscopic evaluation using live/dead staining after 24 h of incubation (Figure 4C,E). On the Zn alloy samples, most cells were dead and had detached from the test surface. The remaining cells exhibited morphological changes, such as cytoplasmic and nuclear condensation. These cells appeared spherical and showed yellow or red coloration due to the dye, indicating damaged cell membranes. In contrast, a dense cell layer had formed on the Ti control surface. The live-dead staining was conducted solely for quality assurance and visual assessment of cell morphology. A more precise quantitative analysis was performed using the BrdU test.

Additionally, cells growing in the well plate in direct proximity to the L-PBF-manufactured alloys also exhibited poor or no growth. Again, morphologically, these cells appeared smaller and rounder, lacking the stretched-out appearance seen in the control. An inhibition zone was visible (Figure 4A(a)). A cell layer was detectable only at some distance from the plates, becoming denser with increasing distance (Figure 4A(b)). The inhibition zone around the Cu plate was also clearly visible, with cells growing only at some distance from the test plates in a dense layer. No inhibition zone was present in the Ti negative control, where cells grew uniformly across the entire well, exhibiting a density comparable to the control well without test plates.

#### 3.2.2. Cytocompatibility Extract Testing

To assess the potential cytotoxic effects of L-PBF-manufactured Zn parts and to identify any differences among the various alloys, a cytocompatibility extract test was conducted at multiple dilutions. For this purpose, L929 and SAOS-2 cells were cultured for 24 h in three different extract concentrations: undiluted (150 µL extract), diluted 1:3 (50 µL extract) and diluted 1:10 (15 µL extract). Cell proliferation was then assessed using BrdU ELISA. The results are shown in Figure 4B,D.

Undiluted extracts from all sample plates showed reduced proliferation on both L929 and SAOS-2 cells (Figure 4B,D). The positive Cu control exhibited low proliferation rates of 9.6 ± 4.5% for L929 and 37.5 ± 5.1% for SAOS-2, compared to cells in pure culture medium (100%). The negative Ti control showed proliferation values similar to those in pure culture medium (108.3 ± 13.6% for L929 and 96.8 ± 5.3% for SAOS-2).

When the extract was diluted 1:3 with culture medium, the ZnAgCuMn alloy showed cell proliferation of 8.9 ± 3.4% for L929 and 9.19 ± 5.2% for SAOS-2 cells. Diluted extracts of the other alloys showed no significant proliferation in either cell line. SAOS-2 cells cultivated in diluted Cu extract had proliferation rates of 80.3 ± 12.8% for L929 and 95.1 ± 4.9% for SAOS-2. The Ti control showed no difference from controls without extract (Control: 100% vs. Titanium: 111.1 ± 17.9% for L929 and 103.3 ± 6.5% for SAOS-2).

The 1:10 diluted extract of the ZnAgCuMn alloy again led to the highest proliferation rate among the alloy extracts in both cell lines (ZnAgCuMn: L929: 27.7 ± 12.1%, SAOS-2: 77.9 ± 13.9%; ZnAgCu: L929: 10.7 ± 7.0%, SAOS-2 12.3 ± 3.0%; ZnAgCuTi: L929: 10.4 ± 4.0%, SAOS-2 26.5 ± 19.0%). The proliferation of SAOS-2 cells with extracts of Cu as well as Ti was comparable to the control.

#### 3.2.3. Time Dependency of Extract Cytocompatibility and Ion Release

1. Cytocompatibility

**Figure 5:**
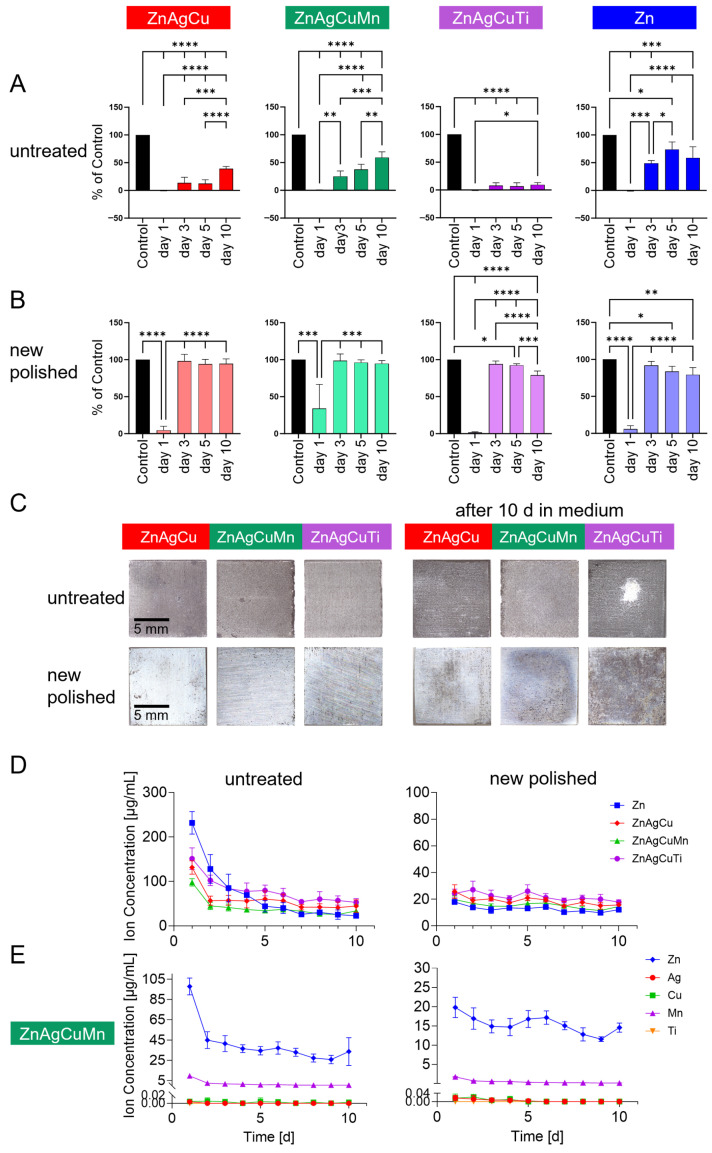
Time dependency of ion release and cytocompatibility of undiluted extracts tested for 24 h with SAOS-2 cells from untreated (**A**) and polished (**B**) L-PBF-manufactured plates. Extracts collected daily over a period of 10 days. (**C**) OM of untreated and polished plates before and after 10 days of immersion. The mean proliferation values with the corresponding standard deviation of independent experiments (*n* = 4) are plotted graphically. All results were normalized to the control cells grown in cell culture medium without extract (=100%). Significance was determined using one-way ANOVA followed by a Tukey test (* *p* < 0.05; ** *p* < 0.01; *** *p* < 0.001; and **** *p* < 0.0001). (**D**,**E**) ICP-OES ion concentration measurement. (**D**) Zn^2+^ release of the different alloys into daily renewed McCoy’s medium from day 1 to day 10. The extracts were taken from untreated and freshly polished samples. The mean values with the corresponding standard deviations of five independent experiments are plotted. (**E**) Ion concentrations of Zn^2+^, Ag^+^, Cu^2+^, and Mn^2+^ release of ZnAgCuMn into the daily changed McCoy’s medium (*n* = 5).

Understanding the kinetics of ion release is essential for evaluating the cytocompatibility of the investigated alloys. To achieve this, the extract medium of L-PBF-manufactured samples from both untreated and freshly polished plates was renewed daily, with measurements taken for proliferation and ion release determination. Undiluted extracts collected at 24 h, and after 3, 5, and 10 days, were added to cultured SAOS-2 cells (Figure 5A,B), and proliferation was measured after 24 h. Figure 5C displays images of the sample plates before and after 10 days of extract preparation.

The cytocompatibility of untreated ZnAgCu and ZnAgCuMn alloys, as well as Zn extracts, decreased over time, with ZnAgCuMn showing the lowest cytocompatibility. A significant increase in proliferation was observed by day 3 (ZnAgCu: 13.5 ± 10.3%, ZnAgCuMn: 25.2 ± 9.7%, Zn: 49.1 ± 4.9%), reaching 39.1 ± 4.2% for ZnAgCu, 59.1 ± 10.1% for ZnAgCuMn, and 58.9 ± 19.9% for Zn by day 10. The untreated ZnAgCuTi alloy was an exception, exhibiting consistent low proliferation rates throughout the period (day 3: 7.7 ± 5.2%, day 10: 9.4 ± 3.8%). However, even after 10 days, none of the untreated alloys reached the values of the control group (Figure 5A).

On polished surfaces (Figure 5B), the undiluted extract of the alloys showed increased cytocompatibility from day 3 onward, achieving results comparable to the control without extract (100%) (day 3: ZnAgCu: 98.0 ± 9.1%; ZnAgCuMn: 98.6 ± 9.0%; ZnAgCuTi: 94.1 ± 4.1%). However, the difference from the control group remained significant for the ZnAgCuTi alloy.

2. Ion release

To examine the changes in ion release over time in McCoy’s medium and to assess the impact of surface conditions on this release, extracts were collected daily over a 10-day period, with ion release measured each day.

As illustrated in Figure 5E, Zn^2+^ release was highest on day 1 with untreated plates and decreased significantly over the sample incubation period. This effect was most pronounced in pure Zn samples, where the Zn^2+^ concentration dropped significantly from 231.54 ± 25.26 µg/mL on day 1 to 22.60 ± 2.09 µg/mL on day 10. The ZnAgCuMn alloy exhibited the lowest Zn^2+^ release on the first day (day 1: 97.75 ± 8.26 µg/mL; day 10: 33.57 ± 13.75 µg/mL). Among the samples, the Zn alloy with titanium showed the highest Zn^2+^ release from day 3 onward, reaching a concentration of 52.91 ± 8.54 µg/mL after 10 days.

The release of Zn^2+^ in new polished samples was significantly lower than in untreated samples by a factor of 12 for Zn on day 1 (Zn untreated: 231.54 ± 25.26 µg/mL, Zn new polished: 17.94 ± 1.19 µg/mL). There were no differences between the various new polished alloys on any day. The release of Zn^2+^ decreased slightly for all alloys over the 10 days (day 1: ZnAgCu: 25.80 ± 5.11 µg/mL, ZnAgCuMn: 19.77 ± 2.65 µg/mL, ZnAgCuTi: 24.31 ± 3.24 µg/mL; day 10: ZnAgCu: 15.73 ± 1.69 µg/mL, ZnAgCuMn: 14.55 ± 1.20 µg/mL, ZnAgCuTi: 17.84 ± 1.71 µg/mL).

After 24 h, small amounts of copper were detected in the extracts of all samples. The concentration was lowest in the ZnAgCuMn and highest in the ZnAgCuTi samples (untreated: ZnAgCu: 0.0192 ± 0.0055 µg/mL, ZnAgCuMn: 0.0046 ± 0.0049 µg/mL, ZnAgCuTi: 0.0273 ± 0.0053 µg/mL). There was no significant difference in the Cu^2+^ concentration in the extracts between the untreated and new polished samples after 24 h. The Cu^2+^ release decreased to zero on day 5 except for ZnAgCu new polished: 0.0019 ± 0.0044 µg/mL, ZnAgCuMn untreated: 0.0041 ± 0.0091 µg/mL, ZnAgCuTi untreated: 0.0256 ± 0.0056 µg/mL. Due to the higher electrochemical potential compared to Zn, the release of Ag and Cu is much slower.

Manganese in ZnAgCuMn alloy was released in the untreated samples at a concentration of 9.57 ± 2.41 µg/mL on day 1 until a value of 0.43 ± 0.04 µg/mL was reached on day 10. The new polished samples released 1.78 ± 0.09 µg/mL on day 1 and 0.16 ± 0.04 µg/mL on day 10 (Figure 5E).

No titanium and silver ions were detected in any extract of any alloy at any time.

In conclusion, the extract of the newly polished Zn alloy with manganese exhibited the lowest Zn^2+^ release on all measured days compared to all other alloys. In the cytotoxicity extract test, the newly polished ZnAgCuMn alloy demonstrated the least toxicity after 24 h of incubation.

3. Surface characteristics after immersion

**Figure 6:**
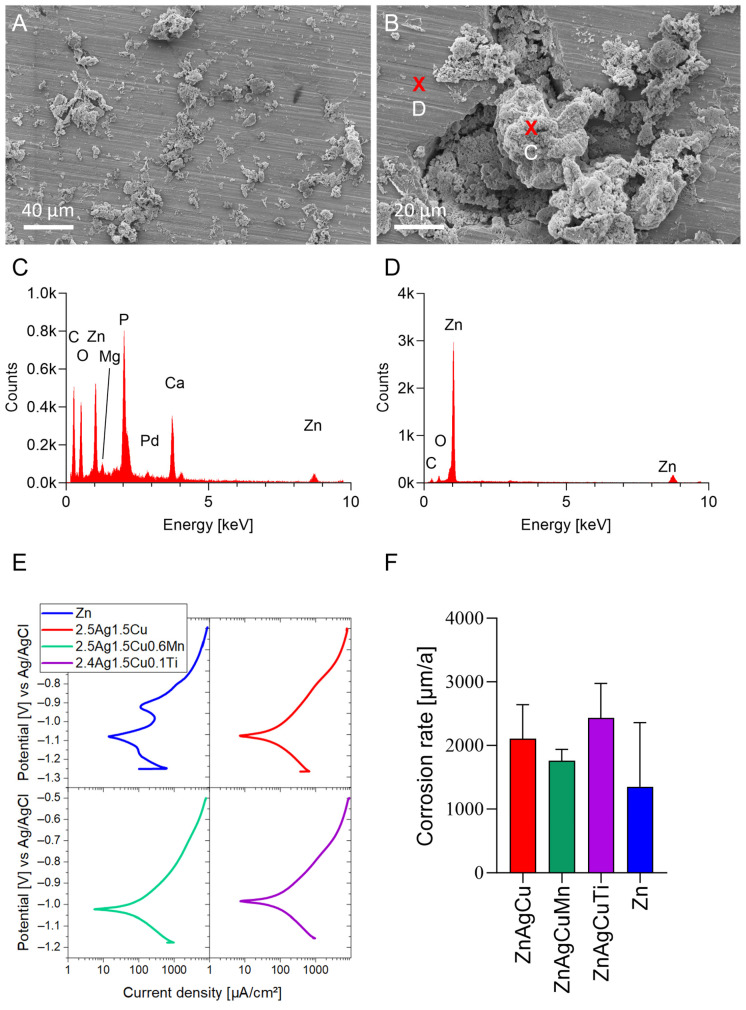
SEM and EDS analysis (**A**–**D**) of newly polished ZnAgCuMn alloy after immersion for 10 days in McCoy’s cell culture medium. (**A**) Overview of the surface with deposits. (**B**) Higher magnification section of the crystalline deposit with EDS spots (red cross) (**C**) EDS spectrum recorded directly at the site of crystalline deposits as indicated in the corresponding SEM image. (**D**) The EDS spectrum measured next to the deposits. (**E**) Potentiodynamic polarization curves calculated from five measurements, respectively. (**F**) Corrosion rates (µm/a) of the untreated samples in PBS supplemented with 10% FBS, calculated from potentiodynamic polarization curves by Tafel extrapolation (*n* = 5).

Scanning electron microscopy of the surfaces of all alloys after 10 days of storage in McCoy’s medium showed crystals formed on the samples (exemplarily shown are the corrosion products on the ZnAgCuMn alloy in Figure 6A,B,C). In addition to Zn, EDS analysis of these corrosion products revealed significant levels of calcium, phosphorus, carbon, and oxygen. These elements were absent in areas without crystal formation (Figure 6D) and on samples that had not been immersed.

### 3.3. Potentiodynamic Polarization

The corrosion rate (Figure 6E,F) reflects the kinetics at which a biodegradable implant dissolves due to corrosion in a physiological solution. This measurement is crucial because the implants need to provide mechanical support until the body has regenerated its own bone. Various factors can influence the corrosion rate, including the addition of different metals and the concentration of these metals within the alloy. In this study, the corrosion rates for the untreated samples of the tested alloys were determined by software using Tafel extrapolation as follows: Zn 1352 ± 1008 µm/a, ZnAgCu 2104 ± 539 µm/a, ZnAgCuMn 1761 ± 176 µm/a, and ZnAgCuTi 2431 ± 544 µm/a (Figure 6F). These results show tendencies, but no statistically significant differences between means.

### 3.4. Influence of Aging on the Cytotoxicity

**Figure 7:**
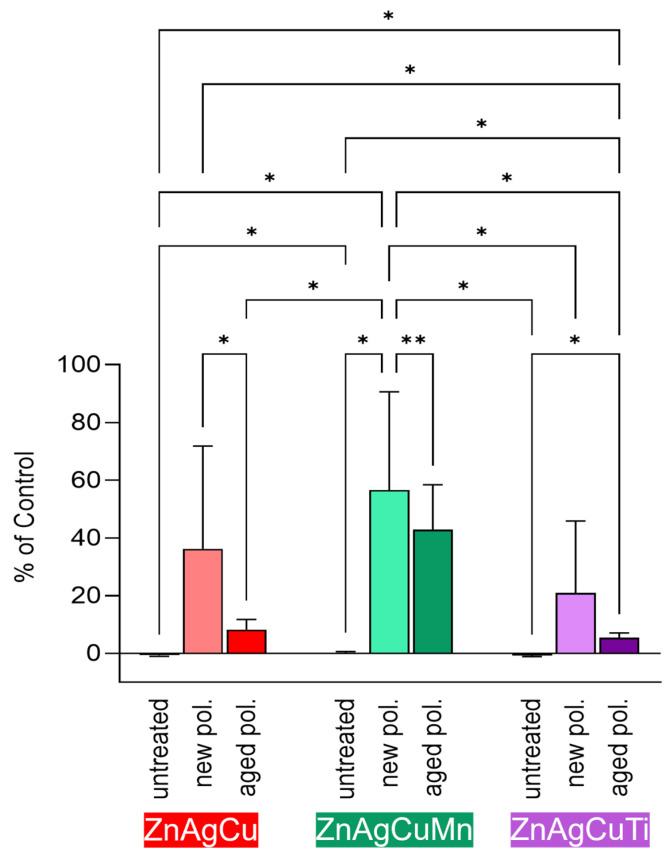
Cytocompatibility extract test with SAOS-2 cells cultivated for 24 h in extracts from untreated, new polished and aged polished samples. The mean proliferation values with the corresponding standard deviation of independent experiments (*n* = 8) are plotted graphically. All results were normalized to the control cells grown in cell culture medium without extract (=100%). Significance was determined using one-way ANOVA followed by a Tukey test (* *p* < 0.05; ** *p* < 0.01).

To evaluate the impact of aging of Zn alloys on SAOS-2 osteoblast cytocompatibility, untreated L-PBF-manufactured samples were compared with freshly polished and aged polished samples using the extract test. After 24 h, undiluted extracts from aged Zn alloys led to reduced osteoblast proliferation compared to extracts from freshly polished plates (Figure 7), highlighting the significant influence of surface aging on cytocompatibility.

SAOS-2 cells exposed to extracts from freshly polished surfaces showed proliferation rates of 36.2 ± 35.5% for ZnAgCu, 56.6 ± 33.9% for ZnAgCuMn, and 20.9 ± 24.9% for ZnAgCuTi relative to controls in pure cell culture medium. In contrast, cells exposed to extract medium obtained from aged surfaces achieved only 8.3 ± 3.5% for ZnAgCu, 42.9 ± 15.6% for ZnAgCuMn, and 5.5 ± 1.5% for ZnAgCuTi (untreated samples: 0 ± 0.3% for ZnAgCu, 0.1 ± 0.6% for ZnAgCuMn, and 0 ± 0.4% for ZnAgCuTi). Among the investigated alloys, ZnAgCuMn demonstrated the highest proliferation when freshly polished, while ZnAgCuTi consistently showed the lowest proliferation values across both surface conditions. Notably, cell proliferation was significantly higher for extracts from newly polished ZnAgCu and ZnAgCuMn samples compared to their aged counterparts. For ZnAgCuTi, however, no statistically significant difference in proliferation was observed between newly polished and aged surfaces. Additionally, proliferation values for newly polished ZnAgCuMn were significantly higher than those for newly polished ZnAgCuTi, whereas the comparison with newly polished ZnAgCu revealed only a non-significant trend.

## 4. Discussion

Zn is considered a promising biodegradable material for implants due to its natural presence in the body, favorable biocompatibility, and tunable corrosion properties, leading to extensive research for biomedical applications.

Three distinct L-PBF-manufactured Zn alloys (ZnAgCu, ZnAgCuMn, and ZnAgCuTi), each surface modified by different treatments, respectively, were characterized and assessed for cytocompatibility using the mouse fibroblast cell line L929 and the human osteoblast cell line SAOS-2. In this study, the investigated alloys were produced exclusively by L-PBF. Comparable alloys manufactured by conventional processing routes were not included. Therefore, a direct comparison of the biological response between additively manufactured and conventionally processed Zn alloys was beyond the scope of the present work.

### 4.1. Powder Production and L-PBF

Quantitative chemical analyses of the alloy (Table 2) after each processing step revealed a pronounced increase in the contents of the alloying elements Ag, Cu, Mn, and Ti during atomization attributed to powder melting with a high-energy laser during part manufacturing. This effect is due to the considerably lower melting and evaporation points, respectively, of Zn compared to the alloying elements, causing excessive evaporation.

As-printed L-PBF-parts are characterized by a high surface roughness compared to conventionally manufactured parts (Figure 3A and Table 3). Due to the steep temperature gradients and rapid solidification in L-PBF manufacturing, the microstructure of the resulting parts is free of a second phase (Figure 3B). Notch-like pores were observed at the surface, which can adversely affect the mechanical performance through the initiation of cracks. Furthermore, partially melted powder particles are usually attached to the surface. While confocal microscopy provides precise roughness mappings of the surface, optically hidden structures, like certain types of pores, are not detectable. Therefore, the box counting method was employed to derive the fractal dimension from µCT data. A discrepancy between the two approaches has been observed, i.e., the order of the roughness (Sa) is ZnAgCuTi < ZnAgCuMn < ZnAgCu, whereas the arrangement of the fractal dimension is the exact opposite, ZnAgCu < ZnAgCuMn < ZnAgCuTi. Although the small sample sizes may have affected the outcome (three vs. five for each alloy), the trends suggest that attention must be paid to such porous surfaces.

Laser melting of a contour line in each powder layer proved to have a sealing effect, i.e., the boundary zone tends to be denser than the core (Figure 2). Regarding Zn alloys, the process window is very narrow, and heat accumulation and impaired heat dissipation, respectively, in the core region of parts during L-PBF production can easily lead to increasing porosity [54]. However, the polished surfaces are similar in terms of smoothness and density, allowing for a direct comparison of the effects of composition, microstructure, and aging.

### 4.2. Cytocompatibility

#### 4.2.1. Direct and Indirect Cytocompatibility Tests

Many in vitro studies have reported that Zn and its alloys are generally well-tolerated as implant materials [59,60,61,62,63,64,65,66,67]. Still, comparing cytocompatibility results across studies is challenging due to variations and differences in the manufacturing methods of the samples, the experimental design, including differences in concentration of the extracts tested, sample size, temperature, and duration of extract preparation.

In this study, undiluted extracts from all tested L-PBF-manufactured Zn alloys exhibited cytotoxic effects with minimal cell survival after 24 h (Figure 4). Correlation between dilution of the extract and improved cell viability is consistent with literature reporting good cellular tolerance for diluted extract [39,68,69]. However, within a physiological context, cells in the vicinity of an implant come into direct contact with the material’s surface without dilution. Therefore, this study also examined the cytocompatibility of undiluted extracts and direct cell contact.

As noted by Watroba et al., some in vitro studies used tetrazolium-based assays, such as MTT, CCK-8, and XTT, to determine cell viability. However, the interaction of Zn^2+^ ions with tetrazolium salts can cause the transformation into formazan, potentially yielding false-positive cell viability results [61]. To avoid this interference, BrdU staining was used in this study to measure cell proliferation. This choice of method could potentially account for the observed reduced proliferation of undiluted extracts in this study compared to tetrazolium-based assays of others.

More recent in vitro studies, however, report cytotoxicity levels similar to those observed in our study [70]. Lietaert et al. also found low cell viability of the tested mesenchymal stem cells on their L-PBF-produced scaffolds [71]. Ramirez-Ledesma et al. demonstrated that undiluted extracts from two different ZnAgMg alloys exhibited significant cytotoxicity towards HUVEC cells in in vitro experiments. Acceptable biocompatibility was observed only at dilutions of 10-fold and 100-fold [72]. The study by Huang et al. [43] also highlights that diluted extracts show better cell viability. Live/dead staining showed that most MC3T3-E1 cells were alive after 1 day of culture in the 10%, 20%, and 50% diluted extracts. However, only a few viable cells were observed in the 100% extracts, which is consistent with the results of this study, where almost no surviving cells were found on the test surfaces, and notable cell survival was observed only with diluted extracts (Figure 4).

SEM imaging from Watroba et al. revealed poor cell attachment on Zn and Zn-3Ag disks [61]. Slightly more cells adhered to the Zn-3Ag-0.5Mg alloy surface, which may be attributed to a lower release of Zn^2+^ ions or factors related to the alloy’s microstructure and surface chemistry. Yet, the cells observed on the Zn-3Ag-0.5Mg alloy had a globular morphology, suggesting cell death. The data showed that high local concentrations of Zn^2+^ ions released from the samples contributed to cell mortality. These findings were in accordance with the observations made in the present study, where direct cytocompatibility testing showed broad inhibition zones surrounding the test plates and morphological changes indicating cellular distress (Figure 4). SEM imaging also showed no vital cells on the alloy surfaces.

Studies have also shown that tolerable Zn ion concentrations in the cell culture medium vary significantly with cell type, as each type displays a unique sensitivity to Zn^2+^. For instance, studies by Kubásek et al. reported acceptable cytotoxicity levels for Zn^2+^ on U-2-OS cells, while L929 cells were more sensitive [73]. Shen et al. showed that the human osteosarcoma cell line HOS was much more sensitive to extracts of Zn-1.2Mg alloys compared to MG63 cells [63]. Consistent with this, the two cell lines used in this study (SAOS-2 and L929) exhibited varying sensitivities to Zn. While both cell types showed adverse responses to the extracts, L929 mouse fibroblasts were more sensitive than human osteoblasts (SAOS-2) (Figure 4).

#### 4.2.2. Influence of Extraction Medium

The cytotoxicity of Zn alloys is also highly dependent on the extract medium, as corrosion behavior and the concentration of released corrosion products vary with the specific environment [74,75]. FBS-supplemented media accelerate the degradation process [76], increasing ion release into the medium. Released metal ions are probably bound by FBS proteins, altering their solubility and bioavailability. However, opposite effects of proteins have also been reported. Pre-incubation of ZnMg alloys in simulated body fluid has also been shown to decrease corrosion rates and improve cytocompatibility [77]. Albumin adherence to alloy surfaces during early degradation stages can induce passivation, slightly reducing corrosion rates [78]. Therefore, the quantification of ion release is essential for understanding cytocompatibility. In this study, the cell culture medium with 15% FBS was deliberately chosen as the extraction medium to capture with physiological relevance the actual ion release that cells would experience. Its high FBS content might also have contributed to the elevated ion release and reduced proliferation observed in undiluted extracts (Figure 5).

#### 4.2.3. Influence of Surface Area, Ion Release and Corrosion

Ion release is heavily influenced by surface structure and chemistry. Untreated L-PBF surfaces, with their high roughness and porosity, released more ions than polished samples (Figure 5). Typically, studies use extracts from plates with a surface area between 1 and 3 cm^2^/mL of medium, but rarely specify sample roughness. In this study, the plates had a geometric surface area of approximately 3 cm^2^, without accounting for additional roughness. Considering surface roughness and porosity, the real surface area is a multiple of that, by a factor of about 4.4 (Table 3). This surface enlargement facilitated greater ion diffusion into the solution, thereby contributing to the observed cytotoxicity (Figure 5). Lietaert et al. investigated L-PBF-manufactured porous scaffolds and likewise found that the true surface area plays a crucial role. Zn^2+^ release correlated with the surface area and thus with the toxicity for mesenchymal stem cells [71].

The rough and undercut topography of the test plates used for this study prevented accurate quantification of their true surface area using optical measurement methods. To standardize conditions, the plates were polished to achieve a defined, measurable surface. In a 10-day long-term experiment, the extract medium was renewed daily to mimic in vivo conditions, where dissolved ions are continuously cleared by blood and lymphatic circulation. The resulting ion concentration profile demonstrated an initially rapid corrosion rate, which progressively declined over time as surface corrosion products formed. This phenomenon can be attributed to two key mechanisms. Zinc is known to rapidly form corrosion products, such as zinc oxide, zinc hydroxide, and basic zinc salts in aqueous environments. These layers can partially passivate the surface and reduce further ion release. Previous studies on biodegradable Zn alloys have shown that such layers can significantly slow down corrosion kinetics after the initial exposure period. As also suggested by the EDS results shown in Figure 6, the formation of calcium- and phosphate-containing deposits may occur on the alloy surface during immersion in DMEM. Such mineral layers are commonly reported for biodegradable metals and may further limit metal ion release [30].

Untreated plates exhibited significantly reduced proliferation compared to polished samples, consistent with ion release data (Figure 5). These ICP-OES measurements of released ions correlated with cellular responses, and Zn^2+^ release was markedly elevated in untreated samples during the first 24 h, likely due to their larger effective surface area and potential pitting corrosion—both of which align with the observed reduction in proliferation (Figure 5).

Studies indicate that high Zn^2+^ concentrations (5 μg/mL) can reduce cell adhesion and proliferation and inhibit osteogenic differentiation [79]. However, findings are inconsistent; while some studies report that Zn^2+^ concentrations above 1 μM inhibit osteoclastogenesis, others note similar effects at sub-nanomolar levels [15]. It is evident that in this study the extracts of untreated Zn alloys from the first 24 h exhibit a toxic effect on the cells, caused by the release of Zn^2+^ exceeding 150 µg/mL. About 20 µg/mL from day 3 onwards is well tolerated by the cells. ICP-OES measurements showed that Zn^2+^ release was lowest in the “new” polished ZnAgCuMn alloy compared to other alloys, which likely contributed to its favorable performance with SAOS-2 cells. In addition, a low concentration of Mn^2+^ was co-released in the ZnAgCuMn alloys, which may have further enhanced cytocompatibility (Figure 5E). The biocompatible degradation by-products of Zn alloys could support tissue healing and regeneration.

The absence of Cu- or Ag-ions in the measurements is most likely a result of selective corrosion, in which Zn dissolves preferentially due to its lower electrochemical nobility compared to copper and silver. This behavior is analogous to the dezincification in brass. Although increased surface area and porosity can enhance the overall ion release, it does not alter the fact that Zn, being less noble, is more likely to dissolve. It is important to note that silver is notoriously difficult to detect with ICP-OES because it typically forms sparingly soluble salts (e.g., AgCl), which limits its detectability in solution. Thus, even if Ag^+^ ions had been released, they would likely not have been detected by ICP-OES. It is difficult to identify the higher copper concentration in the ZnAgCuTi alloy as one cause of the reduced cytocompatibility. This should be investigated further.

Scanning electron microscopy revealed crystal formation on the Zn alloy surfaces after 10 days in McCoy’s medium (Figure 6A,B). The EDS analysis of these crystals detected Zn, calcium, phosphorus, carbon, and oxygen, absent in crystal-free areas (Figure 6C,D). These findings are consistent with those of Huang et al. [43], who identified similar elements in corrosion products of Zn–Mn alloys in simulated body fluid, suggesting the formation of Zn oxide and hydroxyapatite.

The amount of Zn^2+^ ion release measured by ICP-OES was confirmed by the corrosion rates of the untreated samples for the different alloys, determined with the Tafel extrapolation method (Figure 6E,F). Due to the indeterminate real surface area of the L-PBF-manufactured samples, the corrosion rates determined in this study cannot be directly compared to those reported in the literature. The corrosion rates for Zn alloys reported in the literature vary significantly [20,80,81,82], primarily depending on factors such as the method used, temperature, phases, microstructure, surface area, surface condition, and the medium involved. The corrosion rates measured here are relatively high. This is probably due to the medium containing proteins used for measurements. Comparable corrosion rates to our study have also been observed in the Ramirez-Ledesma potentiodynamic polarization tests [72]. Huang et al. [43] also reported that alloys with higher Mn content exhibited lower corrosion rates, which is consistent with our findings. The limitation of the electrochemical polarization methods is that it determines instantaneous corrosion rates under non-equilibrium conditions due to the applied potential. These methods, therefore, cannot directly represent long-term degradation behavior under physiological conditions. Although electrochemical polarization does not represent long-term degradation kinetics, the very high corrosion current densities measured in our experiments are consistent with the high Zn ion release observed in the biological experiments, suggesting a strong initial dissolution of the alloy surfaces.

Studies found that manganese improves mechanical properties, such as yield strength, ultimate tensile strength, elongation, and hardness after hot rolling. During the L-PBF process, manganese refines grain size and reduces the corrosion rate, extending implant service life. Various Zn alloys containing manganese have demonstrated favorable mechanical and cytotoxic profiles [26,28,36,43,50,83,84,85,86]. Manganese promotes osteoblast adhesion and proliferation, upregulating alkaline phosphatase (ALP) expression, which supports bone formation. Jia et al. showed in an animal model that Zn-manganese alloy implants supported superior osteogenesis, as evidenced by increased new bone formation compared to pure Zn [50]. Cell proliferation and morphology analyses revealed that Zn-Mn alloys exhibit higher cytocompatibility than pure Zn, with added manganese significantly enhancing osteogenic activity, as reflected in ALP activity and osteogenic gene expression [50]. Tzion-Mottye et al. also showed that the addition of Mn to the Zn alloy improves the strength of the material and cell viability of the biodegradable ZnFeMn alloy [85]. Li et al. developed a Zn-0.6Mn-0.05Mg-0.05Ca alloy with the best strength–ductility synergy among Zn–Mn-based alloys, antibacterial behavior and less toxicity towards MC3T3-E1 cells [26]. Consequently, Zn–Mn alloys are promising materials for bone defect treatment and fracture repair due to their outstanding mechanical properties and osteogenic potential. This improvement by adding manganese to the Zn alloy was also demonstrated in this study. Both L929 and SAOS-2 cell tests showed increased cell proliferation during incubation with ZnAgCuMn alloy extracts compared to the alloys without Mn and pure Zn (Figure 4).

While reduced surface area and lower ion diffusion after polishing likely contribute to improved cell proliferation, our findings suggest also that ion release alone cannot fully explain the observed decrease in cytocompatibility.

#### 4.2.4. Influence of Aging on Cytotoxicity

In this study, polished Zn alloys were also subjected to ambient air for three months to facilitate aging. The cytocompatibility of the resulting extracts was compared to that of freshly polished plates. This study shows that cells cultured in aged surface extracts had a lower viability compared to cells subjected to extracts from freshly polished surfaces (Figure 7), which might be explained by differences in surface oxidation levels. When Zn is exposed to air, Zn oxides form on the surface [87], and the amount produced is influenced by factors such as temperature and humidity. Typical oxidation rates of Zn in air range from 0.13 µm per year in dry rural environments to 13 µm per year in more humid industrial conditions. More complex reactions occur in various Zn alloys and across different media [88,89,90]. This is reflected by the formation of Zn oxide, Zn hydroxide, and Zn carbonate corrosion products, though further investigation is needed to confirm this. Other studies indicate that Zn hydroxide and Zn phosphate commonly form on the surfaces of Zn-based implants, influencing cellular function and immune response [91]. Thus, the formation of Zn-oxides and -phosphates may explain the different cytocompatibility observed for the “new” polished and “aged” surfaces in our study.

This suggests that the differences we observed in the proliferation rate cannot be attributed to ion release alone but may also stem from changes in surface chemistry induced by aging (Figure 7). These findings reinforce the idea that cytocompatibility is not governed by a single factor, but rather by a combination of influences—including the aging state of the material itself—which together shape how cells respond.

## 5. Conclusions and Outlook

In this study, we investigated three different Zn alloys (ZnAgCu, ZnAgCuMn, ZnAgCuTi) manufactured via L-PBF, intended for use as temporary implant materials. Before these alloys can be employed as customized biodegradable implants—replacing non-degradable metallic biomaterials, such as titanium or titanium alloys—they must meet specific performance and functionality requirements concerning both bulk and surface characteristics at physiological interfaces. In particular, producing Zn-based implant samples through L-PBF-additive manufacturing that comply with biomedical standards for physico-chemical properties and biological responses remains a significant challenge.

The observed differences in cytocompatibility among the alloys are attributed to the combined effects of factors such as microstructure, corrosion behavior, aging, and chemical composition. While the L-PBF-produced implant samples showed promising results in terms of physical and physicochemical properties, the ions released within the first 24 h clearly induced cytotoxic effects. These effects depended both on the specific Zn alloy and the applied post-processing methods. Notably, newly polished ZnAgCuMn samples demonstrated the most favorable biological responses, thereby motivating further research.

Three key factors likely contributed to the improved cytocompatibility observed in the newly polished ZnAgCuMn samples: (i) a slower release of Zn^2+^ ions, a tendency toward a reduced corrosion rate, (ii) the co-release of Mn^2+^ ions, and (iii) a reduced surface oxidation, which had a positive effect on cell viability. However, the extract from the first 24 h remained cytotoxic due to the elevated release of Zn^2+^ during this initial period. Therefore, developing advanced strategies to mitigate this early ion release is essential.

We have identified post-processing of L-PBF-manufactured Zn alloys as a promising approach to reduce the initial ion release associated with early cytotoxicity. Exploring novel surface modification strategies for L-PBF-fabricated Zn alloys, such as polishing, protective surface coatings (e.g., calcium-phosphate, polymer, oxide coatings [92,93,94,95]) or microstructural optimization, is crucial to control degradation kinetics, minimize early cytotoxic effects, and ultimately improve overall implant biocompatibility to ensure patient well-being.

## Figures and Tables

**Table 1:** Listing of surface treatment of the zinc alloys (sample) and the experiments performed.

Experiments	Cell Lines	Sample Preparation			Results in:
	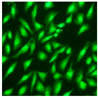	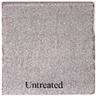	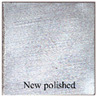	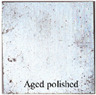	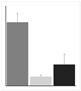
Pore analysis		Untreated			Figure 2A–H
Surface Characterization		Untreated	New polished		Figures 3A–E and 5C
Direct biocompatibility testing	SAOS-2, L929	Untreated			Figure 4A,C,E
Extract testing for biocompatibility (Three extract concentrations)	SAOS-2, L929	Untreated			Figure 4B,D
Extract testing, and time dependency	SAOS-2	Untreated	New polished		Figure 5A,B
ICP-OES		Untreated	New polished		Figure 5D,E
Corrosion		Untreated			Figure 6
Indirect biocompatibility after 24 h, undiluted	SAOS-2	Untreated	New polished	Polished and aged for 3 months	Figure 7

**Table 2:** XRF (X-ray fluorescence spectroscopy) and the ICP-OES analysis of the chemical composition of the ZnAgCu alloy powders (Bal. = Balance).

	ZnAgCu			ZnAgCuMn			ZnAgCuTi		
Element [wt%]	Cast Rod (XRF)	Powder (ICP)	L-PBF Part (ICP)	Cast Rod (XRF)	Powder (ICP)	L-PBF Part (ICP)	Cast Rod (XRF)	Powder (ICP)	L-PBF Part (ICP)
Ag	2.2	3.0	3.1	2.3	2.8	2.8	1.3	1.6	1.7
Cu	1.0	1.9	2.0	1.4	1.6	1.6	2.5	2.8	2.9
Mn	-	-	-	0.6	0.6	0.6	-	-	-
Ti	-	-	-	-	-	-	0.13	0.16	0.17
Zn	Bal.	Bal.	Bal.	Bal.	Bal.	Bal.	Bal.	Bal.	Bal.

**Table 3:** Mean values with standard deviation of the roughness parameters Sa and Sdr from six different locations on three different plates per alloy. The L-PBF-manufactured plates were measured before (untreated) and after polishing (new polished).

Alloy	Sa (Untreated) [µm]	Sa (Polished) [µm]	Sdr (Untreated) [%]	Sdr (Polished) [%]
ZnAgCu	15.3 ± 1.6	0.2 ± 0.2	342.8 ± 44.4	4.0 ± 5.0
ZnAgCuMn	14.4 ± 2.0	0.2 ± 0.1	288.0 ± 30.0	4.9 ± 7.6
ZnAgCuTi	12.7 ± 1.4	0.3 ± 0.2	299.8 ± 39.1	4.9 ± 7.6
Zn (rolled)	0.6 ± 0.2	0.2 ± 0.0	7.8 ± 6.9	3.1 ± 1.0

## Data Availability

The original contributions presented in the study are included in the article, further inquiries can be directed to the corresponding author.
